# Reports of Doses Administered and Adverse Reactions to Snake Antivenom Used in Uruguay in 2018

**DOI:** 10.3389/ftox.2021.690964

**Published:** 2021-08-27

**Authors:** Alba Negrin, María Alejandra Battocletti, Carolina Juanena, Victor Morais

**Affiliations:** ^1^ Toxicology Department, School of Medicine, University of the Republic, Montevideo, Uruguay; ^2^ Department of Biotechnology, Faculty of Medicine, Institute of Hygiene, University of the Republic, Montevideo, Uruguay

**Keywords:** snake antivenom, adverse reaction, safety, bothrops, snakebite accident, effectiveness

## Abstract

In Uruguay, around 60 cases of snakebite accidents occur every year that need to be treated with specific antivenom. They are caused by two snakes of Bothrops genus: *Bothrops alternatus* and *Bothrops pubescens*. Snakebite accidents are mandatory notification events, allowing the acquisition of an accurate registry and a fluent communication with the health care services. The aim of this study is to analyze and report the doses administered to achieve the neutralization of the venom and the adverse reactions caused by snake antivenoms used in Uruguay in 2018, when a change was made in the type of antivenom available. In this year, Uruguay started to use the BIOL® antivenom (lyophilized) and this use coexists with traditional antivenom liquid forms (Vital Brazil and Malbran). The number of patients treated with heterologous BIOL® antivenom were 28 and the ones treated with heterologous solutions Malbran and Vital Brazil antivenoms were 21. The initial dose of BIOL antivenom was 8 vials instead of 4 vials regularly used with the others antivenoms and it achieved the neutralization of most cases (27/28 cases). Early adverse reactions were detected in 4 patients (3 in children) treated with BIOL antivenom and there were no adverse reactions in those treated with Malbran or Vital Brazil antivenoms. Lyophilized antivenom BIOL is being used widely in Uruguay without major complications.

## Introduction

Snakebites by Bothrops genus are the most common snake bite events in Uruguay (Carreira et al., 2008) and it is predominantly an occupational disease in our country rural areas. The Poison Control Centre (Centro de Información y Asesoramiento Toxicológico—CIAT. Toxicology Department Faculty of Medicine) received all consults and advised in the diagnosis, treatment and antivenom administration (WHO, 1988). All the enquiries were registered in the INTOX System, which is an internationally harmonized toxicological Data Management System (DMS) (WHO, 2010a; Morais et al., 2012) and a software system designed for poison centres in the eighties. It enables a poison centre to compile, integrate, and save databases for enquiries, substances, and products. INTOX development is part of the International Programme on Chemical Safety (IPCS)—World Health Organization.

**FIGURE 1:**
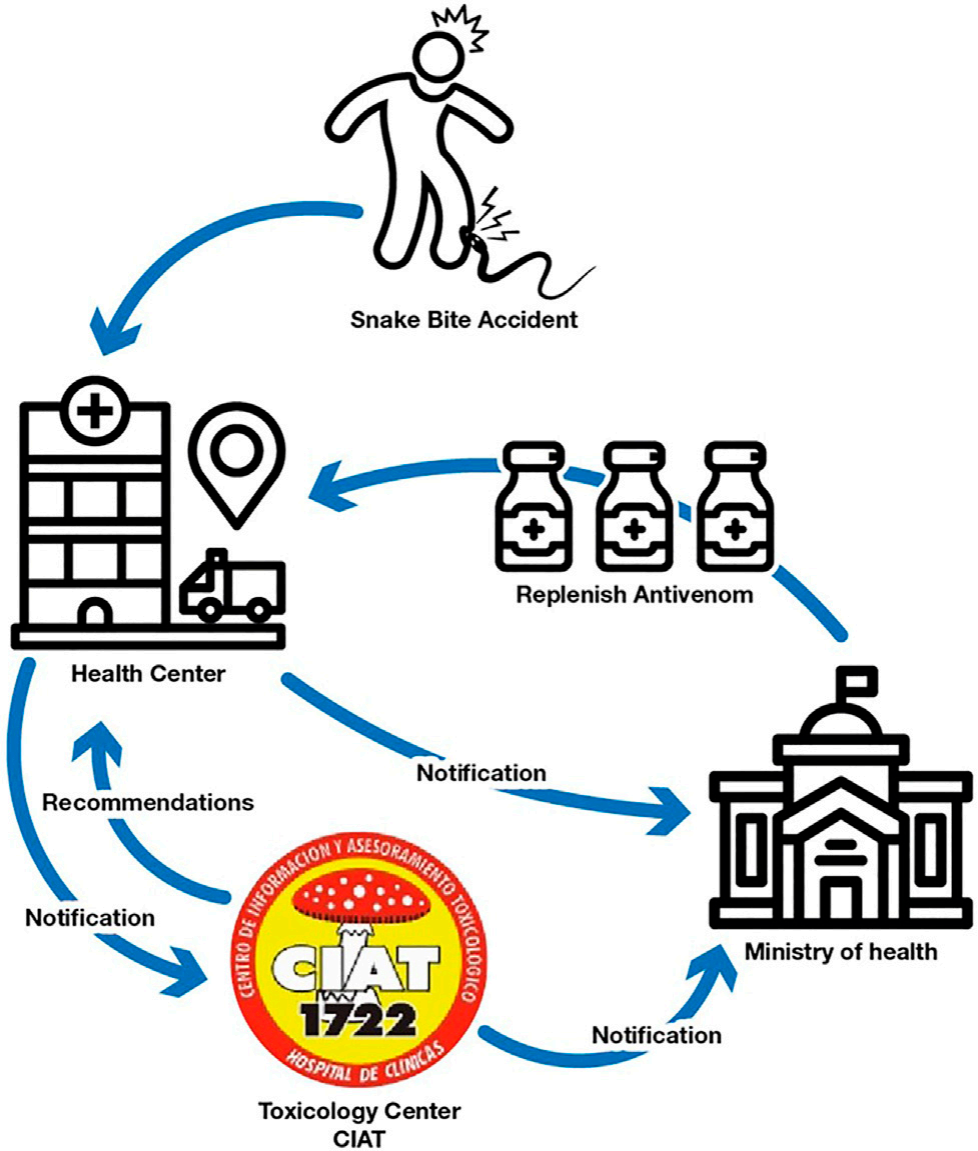
Flowchart of notification steps in a snake bite accident.

Annually CIAT receives over 100 snake bite consults—from which approximately 60 belong to *Bothrops alternatus* and *Bothrops pubescens* accidents—which need to be treated with specific antivenom. The highest incidence of accidents occurs in the summer months and, outside this season, in periods of high temperature and humidity that are recorded in short times in the rest of the year (Carreira et al., 2008). Snakebite accidents are mandatory notification events, allowing the acquisition of an accurate registry of each accident and a fluent communication with the health care services that receive and treat the patients. CIAT professionals follow up the cases and register the antivenom neutralization effectivity as well as possible adverse drug reactions. Figure 1 shows the notification flowchart and the steps that occur when a snake bite accident occurs. The snake antivenom vials are widely spread along health centres in the country and they are distributed exclusively by the Ministry of Health. Underreport is almost not existent because the replacement of the snake antivenom in the treatment health center depends on the case notification.

Bothrops snake venom induces consumption of coagulation factors and important oedema among other effects (França et al., 2003; Serrano et al., 2014; Mamede et al., 2016). Coagulopathy is the major systemic effect of Bothrops envenoming. Antivenoms have been widely used for more than a century for treating snakebites with big success (Chippaux and Goyffon, 1998; Theakston et al., 2003). Unfortunately, using heterologous serum as antivenom make it possible to generate adverse reactions to varying degrees (Morais and Massaldi, 2009; León et al., 2013; De Silva et al., 2016; Morais, 2018). These could be divided from a clinical point of view into two types: early and late reactions. Early hypersensitivity reactions to antivenoms are the major adverse effect of antivenom treatment, including life threatening anaphylaxis. They can start a few minutes after injecting the antivenom endovenously, and range from mild effects such as fever and malaise, to the most severe effects, such as severe hypotension and anaphylactic shock. The causes of these reactions are varied and also depend on the patient’s susceptibility, but generally are due to: activation of the patient’s complement system by the serum antibodies, presence of impurities or aggregates, presence of anti-IgE horse proteins in the patient and currently very infrequently, presence of pyrogens in the antivenom (Morais and Massaldi, 2009; León et al., 2013; De Silva et al., 2016; Morais, 2018). All these causes (except pyrogen reaction) lead to the degranulation of mast cells and basophils causing the release of active compounds, including histamine, that determine different actions such as vasodilation, hypotension, and increased vascular permeability. Late adverse reaction is a type III hypersensitivity, also called “serum sickness”, mediated by antigen-antibody complexes. These complexes lead to complement activation and leukocyte infiltration. Serum sickness occurs from 7 days after the triggering injection, but an accelerated form can occur in subjects who are already sensitized to antivenom (Morais and Massaldi, 2009; Morais, 2018).

Manufacturing protocols and methods of snake antivenoms are different in various regions in the world and the standardization of snake antivenom production remains problematic (WHO, 2010b). They may contain whole immunoglobulins or pepsin or papain digested fragments of immunoglobulins such as F (ab’)^2^ or Fab. Monovalent antivenoms are raised against a single snake species, while polyvalent antivenoms are raised against more than one species. Finally, antivenoms can also be in liquid form or lyophilised (Theakston et al., 2003; WHO, 2010b).

Historically, in our country, antivenom was produced in the Institute of Hygiene between 1990 and 2000 and in 2011. Since then, antivenoms were supplied by regional producers such as Institute Malbran, Institute Butantan, Fundação Ezequiel Dias (FUNED) and Institute Vital Brazil, showing good results of efficacy and security. All of these antivenoms are polyvalent in liquid form and had a potency above 2, 5 mg/ml for both *Bothrops* species. Besides this, all of them are composed by F (ab`)^2^ fragments and are based in ammonium sulphate precipitation protocols with improvements specific to each producer lab (WHO, 2010b). Historical reports of CIAT show that these antivenoms achieved neutralization of between 70 and 85% of the cases with only a single dose (4 vials). The other cases were resolved with double doses (8 vials) and less than 2% needed more doses (12 or 16 vials). Early adverse reaction occurred in less than 10% of patients and in most cases were mild. In 2018, due to a regional shortage of antivenoms, Uruguay started to use a new type of antivenom, Lyophilized Suero Antiofídico Polivalente BIOL®. It neutralizes *Crotalus durissus*, *Bothrops alternatus*, and *Bothrops diporus* but is formulated in lower potency (≥1,5 mg/ml). This antivenom is also composed by F (ab`)^2^ fragment and purification is based in ammonium sulphate precipitation protocol. The aim of this study is to analyze and report the doses administered to achieve the neutralization of the venom and the adverse reactions caused by snake antivenoms used in Uruguay in 2018, when a change was made in the type of antivenom available.

## Methods

A retrospective analysis was made of the cases reported to CIAT in 2018. All the snakebites enquiries were registered in INTOX V4.4 Multi-user using SQL Server 2000 Enterprise.

Clinical case data were obtained from these customized INTOX DMS registries and in the case of missing information, it was requested from the treating hospital. The inclusion criteria consisted of patients who received consultations for a snake bite accident, or had clinical or laboratory evidence of bothropic envenomation and received antivenom. Clinical or laboratory evidence of bothropic envenomation includes patients who have at least one of these characteristics: solenoglyph fangs marks, local pain, oedema and/or ecchymosis in the affected limb, bleeding, gingivorrhagia, alterations in coagulation including long clot time, low prothrombin time, and low fibrinogen quantification. During 2018, 3 types of antivenom were used. One lyophilized (BIOL) and two liquid antivenoms: Vital Brazil (VB) and Malbran (MA). All of them are F (ab`)^2^ fragments and have similar purification processes based in ammonium sulphate precipitation (WHO, 2010b). The declared potency of each producer is shown in Table 1. Each hospital only had one type of antivenom.

**TABLE 1:** Potency and dose used of each group of antivenom.

Antivenom	Potency	Antivenom dose
BIOL	1,25 mg *B*.*alternatus*/ml antivenom	4 vials not used
		8 vials 27/28 (96%)
		16 vials 1/28 (4%)
Malbran	2, 5 mg *B*.*alternatus*/ml antivenom	4 vials 17/21 (81%)
		8 vials 4/21 (19%)
Vital Brazil	5 mg *B.jararaca*/ml antivenom^a^	—

a Vital Brazil antivenom had a ED50 againts B. alternatus venom of more than 2, 5 mg/ml.

The cases were divided in two groups those who received lyophilized antivenom and those who received liquid antivenom. From each group the following variables were analyzed: number and severity of snakebite accidents, antivenom dose used, coagulation parameters restored at 12 h, adverse reactions to antivenom (ADR), severity, and type of the adverse reaction. Cases that presented antivenom adverse reactions were selected and analyzed including: Age, number of vials used, type, and severity of ADR and treatment. Severity and causality of adverse reactions were quantified applying the modified Karch and Lasagna causality algorithm according to the WHO criteria (Karch and Lasagna, 1975; Karch and Lasagna, 1977; Naranjo et al., 1981). Late adverse reactions were not analyzed.

## Results

In 2018 CIAT received 112 consults, of which 109 were clinical cases and 3 were information requests. Bothropic accidents comprised 50 cases and 49 of them required antivenom treatment. All the patients received hydrocortisone as premedication half an hour before antivenom administration. The number of patients treated with heterologous lyophilized BIOL antivenom were 28 and the ones treated with heterologous solutions MA and VB antivenoms were 21. Efficacy results are shown in Table 1. The initial dose of BIOL antivenom was 8 vials instead of 4 vials regularly used with the other antivenoms. Before clinical human use, the potency of antivenoms were tested in mice in order to check the neutralizing dose and confirm cross neutralization with *B. pubescens* (WHO, 2010b). ED50 results confirmed that antivenoms successfully neutralize Uruguayan venoms of *Bothrops alternatus* and *Bothrops pubescens*. ED50 also confirmed that BIOL antivenom vials neutralize over 12, 5 mg of each venom and VB and MA neutralize over 25 mg of each venom in accordance with producers specifications (data not shown). In clinical use, 8 vials of BIOL antivenom achieved the neutralization of most cases (27/28 cases). Only one patient required an extra dose of antivenom (8 vials). In the case of VB and MA, 4 vials were enough to neutralize venom in most patients. However, 4 patients (19%) needed an extra dose 12 h later to complete the restoration of coagulation parameters. Early adverse reactions were detected in 4 patients (3 in children) treated with BIOL antivenom and there were no ADR in those treated with MA or VB antivenoms. Table 2 shows the features of patients that showed adverse reactions and ADR characteristics. All of them received 8 vials (1 dose) of antivenom. The ADR found were compatible to the type of non-IgE mediated anaphylactic reactions (León et al., 2013), and they were catalogued as type B and “Possible” according to the Karch and Lasagna Score. All the adverse reactions were clinically mild and include rush, pruritus, and in one case bronchoconstriction. Patients were successfully treated with antihistamines. None of the patients had previous reports of equine-derived immunoglobulin administration or allergy.

**TABLE 2:** Adverse reaction of antivenoms.

Antivenom	Adverse reactions	Type	Serverity	Clinical manifestation	Score	Treatment
BIOL	4/28 (14%)	Early (4 cases)	Mild (4 cases)	Rush (4 cases)	4 possible (4 cases)	Antihistamines (4 cases; 3 received Chlorpheniramine, 1 received Loratadine)
				Pruritus (2 cases)		
				Bronchoconstriction (1 cases)		
				Cough (1 cases)		
Malbran Vital Brazil	0/21	—	—	—	—	—

## Discussion

The low number of snake bite accidents per year registered in Uruguay is an important limitation of the assay. Some of the conclusions will be confirmed in the next years with the recompilation of more data. Despite this, 2018 was a year where three types of antivenoms coexisted, one of them without clinical experience, and it was a good opportunity to compare them. Lyophilized antivenoms have the advantage that they can be preserves at room temperature and do not need cold chain as liquids antivenoms do. Lyophilized antivenom BIOL was successfully introduced in Uruguay in 2018. Venom neutralization was achieved in all the cases with the adjustment of the administered dose due to its low potency. We observe that 8 vials of BIOL neutralize the venom slightly better than 4 vials of VB or MA. This observation could be explained by the high potency of the used lot. In the case that these results are repeated, a dose adjustment could be considered in the future.

In the past, antivenoms did not exhibit good safety profiles due to the fact that the first antivenoms were poorly purified preparations or even crude sera (Morais, 2018; Squaiella-Baptistão et al., 2018). Over the years, antivenoms began to obtain better safety profiles (Morais and Massaldi, 2009; Squaiella-Baptistão et al., 2018). Currently, antivenom quality varies widely depending on the producer because of the different protocols and manufacturing plant conditions (Morais and Massaldi, 2009; León et al., 2013; Squaiella-Baptistão et al., 2018; Shim et al., 2020). Some antivenoms exhibit adverse reaction rates of less than 10%, while others have values of greater than 50% (Morais and Massaldi, 2009; León et al., 2013; De Silva et al., 2016; Shim et al., 2020). Usually, high incidence of adverse reaction was related to pyrogenic type reactions (Chippaux and Goyffon, 1998). Fortunately, this type of adverse reaction became very unusual in recently years because many producers are implementing strict quality requirements according to good manufacturing practices (GMP) avoiding endotoxin contamination (Morais, 2018).

In Uruguay, records of adverse reactions were under 10% in the last 10 years using liquid forms of antivenoms from Argentina and Brazil. In our study we did not detect any adverse reactions of VB and MA antivenoms. In the case of BIOL antivenom, we found a slight increase in early adverse reactions (14%) but we could only confirm this point in the next years with the compilation of more information. Taking into account that manufacturing processes of assayed antivenoms are similar except the final formulation, we propose that the incorrect solubilization of the antivenom could be causing an increase in the ADR frequency. Beside this, protein aggregates are one of the reported causes of non-IgE anaphylactic reactions like the ones we detected in this work (Morais and Massaldi, 2009; León et al., 2013; De Silva et al., 2016). Medical advice about this point was included in the snake bite protocol with the purpose of decreasing the ADR and confirming this hypothesis in the next few years.

Most of the adverse reactions in this study occurred in children. We did not find any reference or study about this point. However, taking into account that children received the same dose as adults, it could be possible that they have a higher chance of having an adverse reaction. It will be necessary to conduct a recompilation of many years of snake antivenom ADR to confirm this point.

## Data Availability

The original contributions presented in the study are included in the article/Supplementary Material, further inquiries can be directed to the corresponding authors.

## References

[B1] Carreira S. Negrin A. Tortorella M. N. Pino A. Menéndez C. (2008). Ofidismo en Uruguay. Especies Peligrosas y Características del Accidente Ofídico. Montevideo: CID/CEUR. Tradinco.

[B2] Chippaux J.-P. Goyffon M. (1998). Venoms, Antivenoms and Immunotherapy. Toxicon. 36 (6), 823–846. 10.1016/s0041-0101(97)00160-8 9663690

[B3] De Silva H. A. Ryan N. M. De Silva H. J. (2016). Adverse Reactions to Snake Antivenom, and Their Prevention and Treatment. Br. J. Clin. Pharmacol. 81 (3), 446–452. 10.1111/bcp.12739 26256124 PMC4767202

[B4] França F. O. S. Barbaro K. C. Fan H. W. Cardoso J. L. C. Sano-Martins I. S. Tomy S. C. (2003). Envenoming by *Bothrops jararaca* in Brazil: Association Between Venom Antigenaemia and Severity at Admission to Hospital. Trans. R. Soc. Trop. Med. Hyg. 97 (3), 312–317. 10.1016/s0035-9203(03)90158-1 15228251

[B5] Karch F. E. Lasagna L. (1975). Adverse Drug Reactions. Jama. 234 (12), 1236–1241. 10.1001/jama.1975.03260250028021 1242749

[B6] Karch F. E. Lasagna L. (1977). Toward the Operational Identification of Adverse Drug Reactions. Clin. Pharmacol. Ther. 21 (3), 247–254. 10.1002/cpt1977213247 837643

[B7] León G. Herrera M. Segura Á. Villalta M. Vargas M. Gutiérrez J. M. (2013). Pathogenic Mechanisms Underlying Adverse Reactions Induced by Intravenous Administration of Snake Antivenoms. Toxicon. 76, 63–76. 10.1016/j.toxicon.2013.09.010 24055551

[B8] Mamede C. C. N. De Sousa B. B. Pereira D. F. d. C. Matias M. S. De Queiroz M. R. De Morais N. C. G. (2016). Comparative Analysis of Local Effects Caused by Bothrops Alternatus and Bothrops Moojeni Snake Venoms: Enzymatic Contributions and Inflammatory Modulations. Toxicon. 117, 37–45. 10.1016/j.toxicon.2016.03.006 26975252

[B9] Morais V. (2018). Antivenom Therapy: Efficacy of Premedication for the Prevention of Adverse Reactions. J. Venom Anim. Toxins Incl Trop. Dis. 24, 7. 10.1186/s40409-018-0144-0 29507580 PMC5831611

[B10] Morais V. M. Massaldi H. (2009). Snake Antivenoms: Adverse Reactions and Production Technology. J. Venom Anim. Toxins Incl Trop. Dis. 15 (1), 2–18. 10.1590/s1678-91992009000100002

[B11] Morais V. Negrín A. Tortorella M. N. Massaldi H. (2012). Evolution of Venom Antigenaemia and Antivenom Concentration in Patients Bitten by Snakes in Uruguay. Toxicon. 60 (6), 990–994. 10.1016/j.toxicon.2012.07.001 22819686

[B12] Naranjo C. A. Busto U. Sellers E. M. Sandor P. Ruiz I. Roberts E. A. (1981). A Method for Estimating the Probability of Adverse Drug Reactions. Clin. Pharmacol. Ther. 30 (2), 239–245. 10.1038/clpt.1981.154 7249508

[B13] Serrano S. M. T. Oliveira A. K. Menezes M. C. Zelanis A. (2014). The Proteinase-Rich Proteome of *Bothrops Jararaca* Venom. Toxin Rev. 33 (4), 169–184. 10.3109/15569543.2014.922581

[B14] Shim J. S. Kang H. Cho Y. Shin H. Lee H. (2020). Adverse Reactions after Administration of Antivenom in Korea. Toxins. 12, 507. 10.3390/toxins12080507 PMC7472312 32781766

[B15] Squaiella-Baptistão C. C. Sant'Anna O. A. Marcelino J. R. Tambourgi D. V. (2018). The History of Antivenoms Development: Beyond Calmette and Vital Brazil. Toxicon. 150, 86–95. 10.1016/j.toxicon.2018.05.008 29778595

[B16] Theakston R. D. G. Warrell D. A. Griffiths E. (2003). Report of a WHO Workshop on the Standardization and Control of Antivenoms. Toxicon. 41 (5), 541–557. 10.1016/s0041-0101(02)00393-8 12676433

[B17] WHO (1988). The INTOX Data Management System. Available at: https://www.who.int/ipcs/poisons/intox_dms/en/ (Accessed may , 2021).

[B18] WHO (2010a). INTOX Data Management System - Frequently Asked Questions. Available at: https://www.who.int/ipcs/poisons/intox_faqs/en/ (Accessed may, 2021).

[B19] WHO (2010b). WHO Guidelines for the Production, Control and Regulation of Snake Antivenom Immunoglobulins [Internet]. Geneva: WHO Technical Report Series, 135. Available from: http://www.who.int/%0Abloodproducts/snake_antivenoms/snakeantivenomguideline.pdf?ua=1 .

